# A case of herpes simplex virus induced peripheral neuropathy and encephalitis with positive GM3 and CASPR2 antibody

**DOI:** 10.1186/s12883-023-03238-y

**Published:** 2023-05-20

**Authors:** Hongji Lu, Yingdi Liao, Changlin Zhang, Wanxin Wen, Yaming Du, Min Zhao, Lixin Wang

**Affiliations:** 1 The Neurological Intensive Care Unit of Guangdong Provincial Hospital of Chinese Medicine, Guangzhou, 510120 China; 2 The Second Clinical School, Guangzhou University of Chinese Medicine, Guangzhou, 510120 China; 3 The Rehabilitation Department, Kunming Municipal Hospital of Traditional Chinese Medicine, Kunming, 650000 China; 4 The Encephallopathy Department.1 of Guangdong Provincial Hospital of Chinese Medicine, Guangzhou, 510120 China; 5 Department of Stroke Center, The Second Affiliated Hospital of Guangzhou University of Chinese Medicine, Guangzhou, 510120 China

**Keywords:** Herpes simplex virus, Peripheral neuropathy, Encephalitis, GM3 antibody, CASPR2 antibody

## Abstract

**Background:**

We reported on a case involving an older patient with HSV-1 encephalitis who simultaneously experienced the onset of peripheral nerve symptoms associated with the presence of anti-GM3 immunoglobulin G (IgG).

**Case presentation:**

A 77-year-old male was admitted to hospital with high fever, weakness of both lower limbs, and an unstable gait. A CSF test revealed a strikingly increased protein level (1,002 mg/L, normative values: 150-450 mg/L) and MRI revealed hyper-signal lesions in the right temporal lobe, right hippocampus, right insula, and right cingulate gyrus. The CSF was positive for HSV PCR (HSV-1,17870). In addition, the serum samples were positive for CASPR2 antibodies (antibody titer: 1/10) and anti-GM3 immunoglobulin G (IgG) (+). The patient was diagnosed with HSV-1-induced peripheral nerve symptoms that were associated with encephalitis and the presence of anti-GM3 IgG and anti-CASPR2 antibodies. The patient had received included intravenous immunoglobulin, intravenous acyclovir, and corticosteroids therapy. At the one-year follow-up examination, he had regained the necessary skills associated with daily life.

**Conclusions:**

Herpes simplex virus infection often induces encephalitis, and reaction to the virus may trigger an autoimmune response. Early diagnosis and treatment can avoid the progression of the disease to include autoimmune encephalitis.

## Background

The herpes virus can establish a latent infection in the host and cause recurring disease when reactivation occurs. Of the herpes viruses currently identified, the neurotropic herpes simplex virus type 1 (HSV-1) can invade the central nervous system (CNS) and the peripheral nervous system (PNS) [1, 2]. Herpes simplex virus encephalitis (HSVE) is an infectious neurological emergency [3]. HSVE is one of the most devastating viral infections that occur in humans, and the incidence of HSVE worldwide is estimated to be 2 to 4 cases/1,000,000 [3]. 80% of patients with HSVE present with fever, headache, and an altered level of consciousness [4]. Moreover, according to a recent study, 27% of HSVE patients develop autoimmune encephalitis, which primarily results from a potential trigger of the immune response within two months after the infection occurs [5]. However, PNS symptoms resulting from an HSV-1 infection are rare; several studies have indicated that PNS complications of HSV-1 include acute peripheral facial palsy due to reactivation of the HSV, which might be involved in the pathogenesis [1, 6]. In addition, only a series of case reports has documented that some patients with Guillain-Barre syndrome (GBS) have been infected with HSV preceding the onset of acute neurological defects that occurred via a possible mechanism involving an immunological reaction [7, 8]. Therefore, a report was warranted that focused on HSV-mediated encephalitis and peripheral neuropathy. Here, we reported on a case involving an older patient with HSV-1 encephalitis who simultaneously experienced the onset of peripheral nerve symptoms associated with the presence of anti-GM3 immunoglobulin G (IgG). Besides, the virus might have simultaneously triggered an autoimmune response that resulted in the production of positive antibodies against contacting associated protein-like 2 receptor (CASPR2).

## Case presentation

The patient was a 77-year-old male who had not previously experienced small blisters or “blebs” around his mouth, nose, or genitals. He did not have any other diseases. However, he did have a medical history that included intermittent treatment for hypertension. On 9 May 2019, the patient started to suffer from a fever of 38.5℃. He also experienced an aversion to cold, sore throat, painful joints, and a headache with primarily bilateral temporal pain. The patient was treated in the emergency department of the GDTCM. However, his condition continued to deteriorate.

The patient was re-admitted to our hospital on 12 May 2019 due to the presence of a high fever, weakness of both lower limbs, and an unstable gait. After admission, the patient gradually developed weakness in both upper limbs, dysphagia, irritability, loss of comprehension and mental capacity, and disorientation. The patient was transferred to the Neurological Intensive Care Unit (NICU) from the emergency department on 13 May 2019. On neurological examination, the patient exhibited drowsiness, restlessness, decreased short-term and long-term memory, and decreased mental capacity. A cranial nerve examination revealed bilateral peripheral facial palsy, dysphagia, hoarseness, weakness of both sides of the soft palate, and loss of pharyngeal and palate reflexes. His eyes movements were normal. A motor function examination revealed flaccid limb paralysis, loss of tendon reflexes, level 2 muscle strength for both lower limbs, and level 3 muscle strength for both upper limbs. A CSF test revealed a strikingly increased protein level (1,002 mg/L, normative values: 150-450 mg/L) and a red blood cell count of 630/µL (normative values: 0µL ). While the patient’s blood pressure was normal, his glucose, leukocyte, and lymphocyte counts were also normal. The second lumbar puncture revealed an increased protein level (1,745 mg/L, normative values: 150-450 mg/L), a red blood cell count of 43/µL and slight elevated white blood cell (77/µL, normative values: 0–8µL). We initially considered the possibility that the patient had GBS due to the pre-infection, peripheral symmetric damage to his limbs and cranial nerves, and albuminocytologic dissociation. Therefore, we provided intravenous immunoglobulin therapy (IVIG, 0.4 kg/d) for five days.

**Fig. 1:**
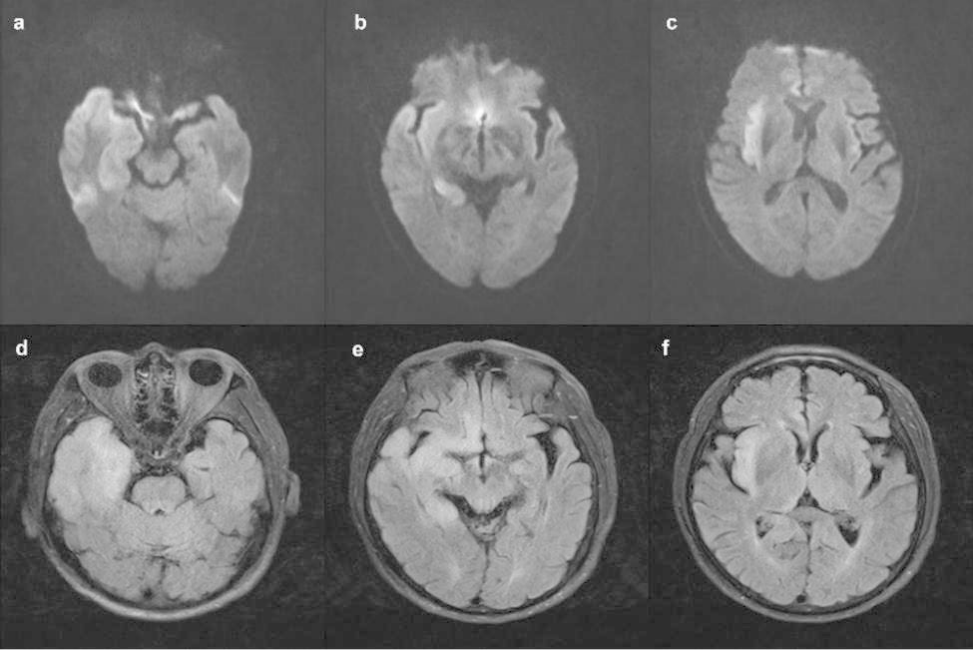
MRIs in acute phase. DWI(**a-c**) and FLAIR(**d-f**) show hyper-signal lesions in the right temporal lobe, right hippocampus, right insula, and right cingulate gyrus

The patient presented a worsening condition one day later, including lethargy, with a GCS score of 7. He developed hypoxemia caused by increased sputum secretion due to bulbar paralysis, which blocked his airway. The patient underwent tracheal intubation and was placed on a ventilator. Subsequently, the patient experienced status epilepticus, and we provided anti-epilepsy treatment. We considered the patient might have experienced CNS damage due to the presence of considerable advanced neurological dysfunction. We carried out MRI imaging and an electroencephalogram (EEG) on the patient. We also performed autoimmune antibody tests on CSF and serum samples. The MRI revealed hyper-signal lesions in the right temporal lobe, right hippocampus, right insula, and right cingulate gyrus (Fig. 1). The EEG revealed epileptiform discharges in the right temporal region and right frontal region, which were consistent with a diagnosis of viral or autoimmune encephalitis. Therefore, the patient was treated intravenously with 10 mg/kg acyclovir every eight hours for eight days. In addition, methylprednisolone (MP, 1 g/d) pulse therapy was administered for five days, followed by a decreased MP dosage (0.5 g/d) for five days, then with oral prednisolone (60 mg/d). During this time, the second-generation sequencing of the CSF sample and the autoantibody spectrum for peripheral neuropathy were evaluated. The CSF was positive for HSV PCR (HSV-1, specific sequence 17,870). The immunologic tests were performed on the serum and CSF samples, including those for anti-ganglioside antibodies associated with peripheral neuropathy and antibodies associated with autoimmune encephalitis. The CSF samples were negative for antibodies of autoimmune encephalitis. The serum samples were positive for CASPR2 antibodies (antibody titer:1/10) and anti-GM3 immunoglobulin G (IgG) (+) using the indirect tissue immunofluorescence and cell-based assays. These tests verified the diagnosis of HSVE but were only suggestive of the presence of autoimmune encephalitis.

**Fig. 2:**
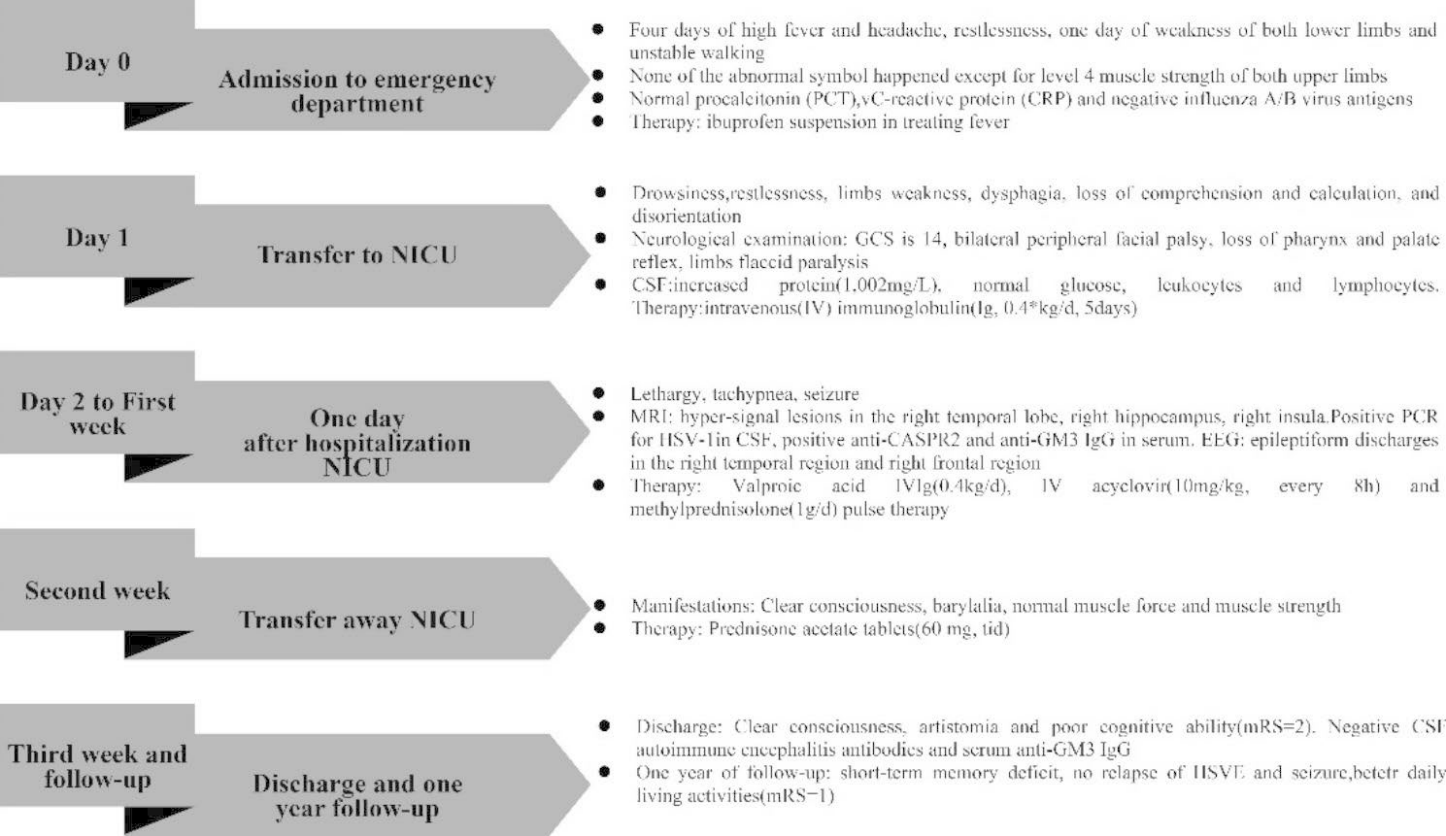
The progress of diagnosis and treatment for this patient

Progressive improvement in consciousness, motor function, and respiratory function was observed during the second week of treatment. The patient was removed from the ventilator and transferred out of the NICU. When the patient was discharged from the hospital, he was completely conscious but had poor cognitive ability (a modified Rankin Scale [mRS] score of 2). The CSF autoimmune encephalitis antibodies and serum anti-GM3 IgG were negative before the patient was discharged. Six months later, this patient presented with fever and delayed reaction was admitted again in GDTCM. His peripheral white blood cells and procalcitonin(2.52 ng/ml, normative values: 0-0.05 ng/ml) were elevated. Chest X-ray showed inflammation of the left upper lingular segment and both lower lungs. There was no abnormal EEG activity. However, the patient refused to undergo a lumbar puncture and we were unable to assess the CSF. On treatment, ceftriaxone, levofloxacin and piperacillin sodium sulbactam were given. Subsequently, the patient’s temperature returned to normal and his mental status improved. At the one-year follow-up examination, despite a deficit in his short-term memory, the patient had not experienced any relapse of the HSVE or occurrence of seizures. He had regained the necessary skills associated with daily life (a mRS score of 1). The whole progress of diagnosis and treatment were shown in Fig. 2.

## Discussion and conclusion

According to preliminary published literature [1], HSV-1 infection can cause not only encephalitis but also peripheral neuropathy [3, 8]. Only a few cases have reported that the clinical features of HSV infection have been identified with other peripheral neurological symptoms such as muscle weakness, except for facial palsy. Previous cases have only reported HSV invasion of the central nervous system alone or the peripheral nervous system alone. Meanwhile, there also is a growing body of literature demonstrating that HSVE may induce autoimmune encephalitis that is primarily mediated by anti-N-methyl-D-aspartate receptors (NMDAR) in the serum and CSF [5]. These results verify that autoimmune antibodies might develop approximately one month after treatment of HSVE.

Here, we report on a case in which an HSV-1 infection induced a peripheral neuropathy syndrome that included progressive limb weakness, cranial nerve defects, and the presence of anti-GM3 IgG in the patient’s serum. Unfortunately, because the patient’s condition worsened, electromyography (EMG) examination was not performed in time. When the patient’s condition improved and was transferred out of the ICU, he underwent EMG examination. The results showed that peripheral damage to the right gastrocnemius nerve (sensory fiber involvement, axonal damage) and prolonged H-reflex latency of the tibial nerve bilaterally. EMG findings do not support demyelination changes,which may be caused by disease progression or drug treatment effect.

Herpes simplex virus (HSV) is one of the common pathogens causing encephalitis. The clinical features of this patient include a history of antecedent infection, acute or subacute onset, mainly manifesting as multiple cranial nerve damage, impaired consciousness, dysphagia, slurred speech. Therefore we have to differentiate it from brainstem encephalitis. And abnormalities associated with HSVE on MRI were seen in the temporal lobe or brainstem [9]. However, this patient’s MRI examination suggested no abnormal signals in the brainstem. In addition, it should be differentiated from limbic encephalitis, which is characterized by memory loss, as well as irritability, confusion, hallucinations, partial or generalized epilepsy, drowsiness, and dementia.Limbic encephalitis refers to the involvement of limbic structures such as the hippocampus, amygdala, insula and cingulate cortex. But in our case, we reported that HSV triggered the production of autoimmune brain CASPR2 antibodies in the serum. Therefore, we assumed that autoimmune antibodies might have appeared in the serum of this older patient after an initial HSV infection. It is well recognized that post-infectious immune-mediated targeting of the nervous system can occur. Infection with HSV triggers a robust immune response that helps clear the infection from the innate immune system. Toll-like receptors generate and activate signaling pathways that result, in the early stages, in the production of pro-inflammatory cytokines such as tumor necrosis factor and various interleukins [10]. The inflammatory process recruits immune cells to induce necrosis and apoptosis of infected cells.

We determined that the patient, who had an HSV infection, experienced both PNS and CNS symptoms and additional examination findings revealed hyper-signal lesions in the right cerebral lobe. A series of complications associated with the PNS occurred that was mediated through the serum peripheral neuropathy antibodies. The question remained as to why there were anti-GM3 Ig-G and CASPR2 antibodies in the patient’s serum. It is well known that anti-ganglioside antibodies can be found in patients with acute inflammatory demyelinating polyradiculoneuropathy. These antibodies might present immunological characteristics, especially antibodies such as GM1, GM1b, or GD1b, which have been observed previously [11]. However, abundant evidence indicates that the expression level of GM3 is negatively correlated with the malignancy of certain types of tumors [12]. We also screened the tumor of this patient, performed the chest CT and abdominal CT, and detected tumor markers,but no tumor was found. Therefore, this is the first report of an older HSVE patient exhibiting anti-GM3 IgG antibodies in association with both lower limb weakness and peripheral cranial nerve damage.

CASPR2 is the primary target antigen for auto-antibodies against the neuronal voltage-gated potassium channel complex, which is expressed and distributed in the CNS and PNS. Anti-CASPR2 could be used as a target of cellular immunity to inhibit effective regeneration of the myelin sheath and serve as a marker for autoimmune encephalitis [13]. However, the CRSPR2 antibody in the CSF was negative, so the patient could not be diagnosed as having autoimmune encephalitis. However, there was a tendency that the condition could have developed into autoimmune encephalitis. The negative results did not rule out the possibility of a timely diagnosis and treatment. In general, HSV-1 might trigger an immune response and produce a series of autoimmune antibodies with peripheral neuropathy presented in HSVE after the first episode of a primary infection. Also, immunocompetent patients exhibit more severe manifestations than older immunocompromised patients [14].

This patient experienced improvement in his neurological impairment, and there was no occurrence of HSVE and epilepsy with the early diagnosis and treatment, given our awareness of the possibility of autoimmune encephalitis. Therefore, it is suggested that when patients with HSVE are admitted, an early test is carried out to look for the presence of autoantibodies, including GM3, CASPR2, and NMDR antibodies in the CSF and serum. Treatment that includes the addition of corticosteroids and immunoglobulin to aciclovir could improve the patients’ prognosis. On the other hand, delaying corticosteroid therapy might prevent the subsequent development of autoimmune antibodies. However, this case study also had shortcomings. We did not retest second-generation sequencing and antibodies associated with autoimmune encephalitis of the CSF sample during follow-up.

The authors would like to express their gratitude to EditSprings (https://www.editsprings.cn ) for the expert linguistic services provided.

## Data Availability

All data related to this case report are documented within this manuscript.

## Abbreviations

CNS: Central nervous system
CASPR2: Contacting associated protein-like 2 receptor
EEG: Electroencephalogram
GBS: Guillain-barre syndrome
HSV-1: Herpes simplex virus type 1
HSVE: Herpes simplex virus encephalitis
IgG: Immunoglobulin G
NICU: Neurological intensive care unit
PNS: Peripheral nervous system
